# Corneal morphological changes after discontinuation of long-term orthokeratology and SMILE surgery outcomes: a retrospective comparative study

**DOI:** 10.1186/s12886-026-04673-4

**Published:** 2026-02-13

**Authors:** Kun Zhou, Xiaohuan Ma, Di Shen, Wenjia Cao, Xiyu Sun, Mengchen Li, Wei Wei

**Affiliations:** 1 Department of Ophthalmology, Xi’an No.1 Hospital, 30 Fenxiang, The South Avenue, Xi’an, Shaanxi Province 710002 China; 2 Shaanxi Institute of Ophthalmology, Xi’an, Shaanxi Province China; 3 Shaanxi Key Laboratory of Ophthalmology, Xi’an, Shaanxi Province China; 4 Clinical Research Center for Ophthalmology Diseases of Shaanxi Province, Xi’an, Shaanxi Province China; 5 The First Affiliated Hospital of Northwestern University, Xi’an, Shaanxi Province 710002 China

**Keywords:** Orthokeratology, Ocular biometry, Keratometry, SMILE refractive surgery

## Abstract

**Background:**

Orthokeratology (OK) has been widely adopted as a non-surgical intervention to slow myopia progression in children and adolescents. In China, where myopia prevalence is exceptionally high, OK lenses are commonly prescribed to school-aged children as an early intervention strategy. As these individuals reach adulthood, many pursue permanent refractive correction—such as small-incision lenticule extraction (SMILE)—to meet unaided visual acuity standards required for university entrance, military service, or certain occupations. However, the long-term morphological effects of OK wear and their potential impact on SMILE outcomes remain inadequately understood. This study aimed to evaluate ocular biometric and corneal morphological changes following long-term OK lens discontinuation and to compare the visual and surgical outcomes of SMILE between former OK users and spectacle-wearing controls.

**Methods:**

This retrospective comparative study included 44 myopic eyes—22 from patients with a history of OK lens wear (average duration: 64.8 ± 22.6 months; discontinuation ≥ 3 months) and 22 from age- and refractive error–matched controls. Examinations were conducted pre-OK (baseline), pre-SMILE, and at 1 month, 6 months, and final follow-up. Parameters included uncorrected and corrected distance visual acuity (UDVA, CDVA), corneal topography, pachymetry, volume, curvature, and higher-order aberrations. Astigmatic outcomes were evaluated using Alpins vector analysis.

**Results:**

After OK discontinuation, the OK group exhibited increased corneal astigmatism (*P* = 0.030), spherical aberration (*P* = 0.036), and total aberrations (*P* = 0.034), as well as decreased vertical coma (*P* = 0.004), central corneal thickness (*P* < 0.001), and corneal volume (*P* = 0.001). Posterior corneal curvature steepened significantly (*P* < 0.001), while anterior curvature remained stable. Further correlation analysis showed that a younger age at initial OK lens wear was significantly associated with greater posterior steepening after discontinuation at the 3 mm, 5 mm, and 7 mm zones (all *P* < 0.050). There was also a borderline negative correlation between the duration of OK lens wear and changes in posterior steep K value, suggesting longer wear may result in more pronounced steepening. At 6 months and final follow-up, both groups demonstrated comparable UDVA, CDVA, corneal shape, and higher-order aberrations (all *P* > 0.050). Vector analysis revealed no significant group differences in astigmatic correction.

**Conclusions:**

SMILE remains a viable option for former OK users; the observed reduction in CCT after lens discontinuation has minimal impact on refractive surgery selection.

## Introduction

Myopia is the most prevalent refractive error globally and has become a major public health concern due to its association with vision-threatening ocular complications​ [1–3]. In East Asia, the prevalence of myopia has escalated dramatically over recent decades, particularly in China, where over 80% of adolescents and approximately 27% of primary school children are affected. The proportion of individuals with high myopia has exceeded 10%, raising concerns regarding long-term visual morbidity and socioeconomic burden​ [4–6]. Orthokeratology (OK) has gained widespread clinical adoption as an effective non-surgical intervention for myopia control in children and adolescents​. This technique involves overnight wear of rigid gas-permeable lenses to induce temporary central corneal flattening, thereby correcting refractive error and reducing axial elongation [7–9]. A recent large-scale cross-sectional survey in China reported a 1.4% (1,021) usage rate of OK lenses among school-aged children, with the majority aged between 10 and 13 years [10].

As these early adopters of OK approach adulthood, many increasingly seek permanent refractive correction, particularly small-incision lenticule extraction (SMILE), to meet the visual standards required for higher education, military service, and occupational requirements ​ [11–14]. However, the long-term effects of OK lens wear on corneal morphology—and their implications for subsequent refractive surgical outcomes—remain insufficiently characterized. Previous studies suggest that corneal shape may largely return to baseline after OK lens discontinuation​ [15]. However, emerging evidence indicates that long-term use may induce subtle, yet persistent structural changes including residual corneal flattening​ [15, 16], increased astigmatism [17], central thinning [18–21], and epithelial remodeling [22, 23].

Given the growing clinical demand for refractive surgery among former OK users, it is critical to understand whether these residual changes affect visual and surgical outcomes following SMILE. The present study aims to investigate ocular biometric and corneal morphological alterations after long-term OK lens discontinuation and to assess their impact on SMILE outcomes compared with eyes of patients without a history of OK wear.

## Methods

### Subjects and inclusion criteria

This retrospective study was conducted in accordance with the tenets of the Declaration of Helsinki and approved by the Ethics Committee of Xi’an No.1 Hospital (Approval number: [2024-008]). Informed written consent was obtained from all patients. A total of 44 eyes of 44 patients were included: 22 patients with a history of long-term overnight OK lens wear (OK group) and 22 age-matched myopic patients who wore spectacles only (control group). All OK treatments and subsequent SMILE procedures were performed at Xi’an No.1 Hospital.

Inclusion criteria required the absence of active ocular disease or injury, no history of ocular surgery, and no use of ocular or systemic medications. Refractive error had to be stable for at least two years prior to surgery (spherical refractive error between − 1.00 D and − 7.50 D). The OK group had worn OK lenses for an average of ~ 5.4 years (64.8 ± 22.6 months) and discontinued lens use for at least 3 months before SMILE​. Only one eye per patient (the right eye) was analyzed to avoid inter-eye correlations​. Corneal stability after OK cessation was confirmed before surgery [24], based on the following criteria:

- Refractive fluctuations within ± 0.50 D over consecutive weekly visits.
- Central and apical corneal curvature variations within ± 0.05 mm.
- Anterior and posterior corneal elevation changes within ± 2 μm.

### Examinations and surgical procedure

All patients underwent comprehensive ophthalmic examinations at multiple time points: pre-OK(before OK lens wear), pre-SMILE (after ≥ 3 months OK cessation for the OK group), 1 month postoperatively, and 6 months postoperatively, with a final follow-up at the last visit (mean 16.50 ± 8.99 months for OK vs. 11.27 ± 2.25 months for controls), the average follow-up time did not differ significantly between the OK and control groups (*P* = 0.201). For the OK group, pre-OK data were also analyzed to evaluate changes after long-term OK use. Uncorrected distance visual acuity (UDVA) and corrected distance visual acuity (CDVA) were measured for each visit and recorded in logarithm of the minimum angle of resolution (logMAR) units. ​Objective and subjective manifest refractions were performed to determine spherical and cylindrical errors. Axial length (Carl Zeiss Meditec AG, Germany) was measured using an optical biometer. Corneal morphology was assessed with a Scheimpflug-Placido tomography device (Sirius, CSO, Italy) at each visit​. Parameters recorded included central corneal thickness (CCT), corneal volume (10-mm central zone), anterior and posterior keratometry (flat K and steep K) at 3-mm, 5-mm, and 7-mm zones, maximum keratometry (Kmax), and corneal astigmatism​. Corneal wavefront aberrations over a 6-mm pupil were recorded, including total corneal aberration, higher-order aberrations (HOAs), spherical aberration, coma (total, vertical, and horizontal), trefoil, and astigmatic aberration components​. Only high-quality topography scans with good centration were accepted for analysis. SMILE surgery was performed using a VisuMax femtosecond laser platform (Carl Zeiss Meditec, Jena, Germany). Laser settings included a cap thickness of ~ 110–130 μm, a 6.5 mm optical zone, and a 2 mm superior corneal incision​. After femtosecond lenticule creation, the intrastromal lenticule was dissected and removed through the small incision​. All surgeries were uneventful and performed by the same experienced surgeon.

### Statistical analysis

Data distribution was evaluated with the Shapiro-Wilk test. Continuous outcomes are reported as mean ± standard deviation. In the OK group, paired comparisons between pre-OK and pre-SMILE values were performed using a Paired t-test for normally distributed data or Wilcoxon signed-rank test for non-normal data​. Between-group comparisons (OK vs. control) at pre-SMILE and post-SMILE time points used an independent-samples t-test for parametric data or Mann-Whitney U test if non-parametric assumptions were met​. Correlation between parameters were determined using Pearson’s correlation tests. Longitudinal changes over postoperative follow-ups were analyzed using general linear mixed models with Bonferroni post hoc correction, and results were summarized as estimated mean differences with standard error, based on previous literature [25]. All statistical analyses were conducted using SPSS version 27 (IBM Corp., Chicago, IL), with a two-tailed *P* < 0.050 considered statistically significant.

## Results

### Patient demographics

A total of 22 subjects (14 females and 8 males) were recruited in the control group, with a mean age of 18.82 ± 1.99 years. The average spherical diopter before SMILE intervention was − 4.23 ± 1.30D. In the OK group, measurements were taken from the right eyes of 22 subjects (11 females, 11 males), with a mean age of 19.18 ± 2.04 years. The mean spherical diopter before OK lens wear was − 3.90 ± 1.69 D. After an average OK treatment duration of 64.77 ± 22.64 months, participants discontinued lens wear for at least 3 months before undergoing SMILE surgery. Average pre-SMILE spherical diopter was − 4.43 ± 1.56 D.

Comparisons of preoperative spherical diopter, age, and corneal curvature between the Control and OK groups are presented in Table 1. No statistically significant differences were observed between the two groups in terms of age, CDVA, spherical diopter and corneal curvature (*P* > 0.050).

**Table 1:** Descriptive statistics (Mean ± SD) for the two groups before SMILE surgery

	*n*	Control SMILE (Mean ± SD)	OK Group (Mean ± SD)	*P* value
Age (years)	22	18.82 ± 1.99	19.18 ± 2.04	0.377^#^
CDVA (logMAR)	22	-0.08 ± 0.04	-0.08 ± 0.04	1.000^*#*^
Sphere (D)	22	-4.23 ± 1.30	-4.43 ± 1.56	0.639*
Cylinder (D)	22	-0.63 ± 0.58	-0.82 ± 0.63	0.268 ^*#*^
Flat K (D)	22	41.96 ± 0.93	42.35 ± 1.29	0.261*
Steep K (D)	22	43.16 ± 0.97	43.74 ± 1.43	0.125*
Corneal astigmatism (D)	22	-1.19 ± 0.54	-1.38 ± 0.80	0.358*

CDVA=corrected distance visual acuity; logMAR = logarithm of the minimum angle of resolution

*Independent sample t-test; ^#^ Mann-Whitney U test

### Corneal morphology changes after OK discontinuation

**Fig. 1:**
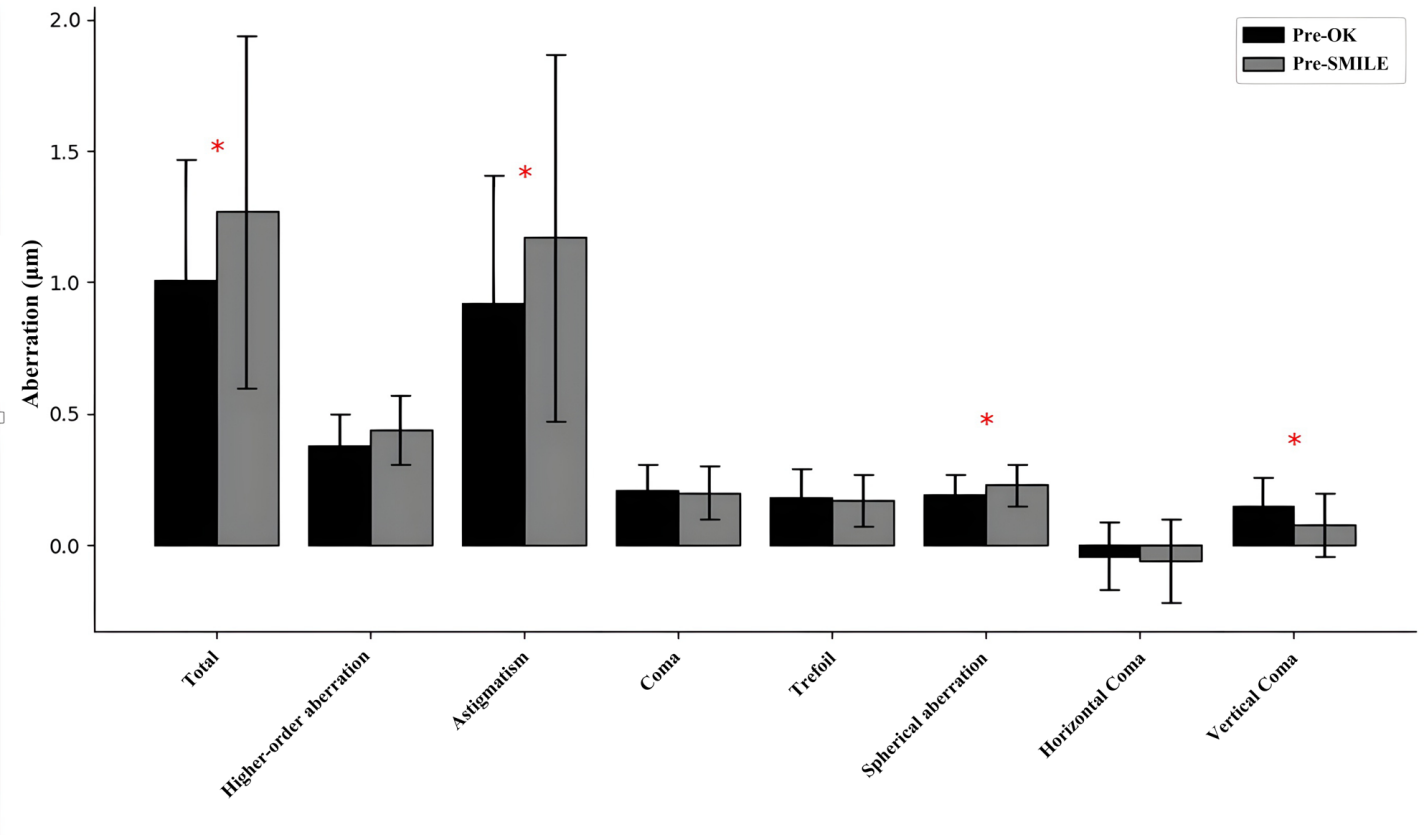
Comparison of anterior corneal aberrations before lens wear (Pre-OK) and prior to SMILE surgery (Pre-SMILE) (Paired Sample t-Test). This bar graph illustrates that total corneal aberration, astigmatism, and spherical aberration significantly increased prior to SMILE surgery (Pre-SMILE) compared to measurements taken before lens wear (Pre-OK) (*P* = 0.034, *P* = 0.037, *P* = 0.036, respectively). In contrast, vertical coma significantly decreased (*P* = 0.004)

Comparing the OK group’s measurements before OK lens wear to those after long-term OK wear and ≥ 3 months cessation (Pre-SMILE), several significant shifts were observed. The manifest spherical power became slightly more myopic, changing from − 3.90 ± 1.69 D Pre-OK to − 4.43 ± 1.56 D Pre-SMILE (*P* = 0.025). Corneal astigmatism increased in magnitude from 1.12 ± 0.46 D to 1.38 ± 0.80 D (*P* = 0.030)​. The cornea exhibited thinning and reduced volume: CCT decreased by ~ 10 μm (from 561.2 ± 6.2 μm to 551.4 ± 6.6 μm, *P* < 0.001) and corneal volume decreased from 60.44 ± 2.92 mm³ to 59.30 ± 3.16 mm³ (*P* = 0.001)​(Table 2). Figure 1 illustrates that total corneal wavefront aberration increased significantly after OK (mean 1.01 μm Pre-OK vs. 1.27 μm Pre-SMILE, *P* = 0.034)​. In particular, astigmatic aberration increased from 0.92 ± 0.49 μm to 1.17 ± 0.70 μm (*P* = 0.037) and spherical aberration from 0.19 ± 0.08 μm to 0.23 ± 0.08 μm (*P* = 0.036)​. By contrast, vertical coma aberration decreased (0.15 ± 0.11 μm to 0.08 ± 0.12 μm, *P* = 0.004)​.

**Table 2:** Comparison of ocular parameters before OK treatment (Pre-OK) and prior to SMILE surgery after at least 3 months of OK treatment discontinuation (Pre-SMILE)

	*n*	Pre-OK (Mean ± SD)	Pre-SMILE (Mean ± SD)	*P* value
Age (years)	22	13.14 ± 2.73	19.18 ± 2.04	<0.001^#^
UDVA (logMAR)	22	0.91 ± 0.34	1.02 ± 0.31	0.171*
Sphere (D)	22	-3.90 ± 1.69	-4.43 ± 1.56	0.025*
Cylinder (D)	22	-0.60 ± 0.43	-0.82 ± 0.63	0.148*
Flat K (D)	22	42.49 ± 1.35	42.35 ± 1.29	0.261*
Steep K (D)	22	43.60 ± 1.44	43.74 ± 1.43	0.296*
Corneal astigmatism (D)	22	-1.12 ± 0.46	-1.38 ± 0.80	0.030*
Kmax (D)	22	44.59 ± 1.72	44.76 ± 1.78	0.526*
Corneal volume (mm^3^)	22	60.44 ± 2.92	59.30 ± 3.16	0.001*
Axial length (mm)	22	25.38 ± 0.92	25.57 ± 0.78	0.114^*#*^
CCT (µm)	22	561.23 ± 6.18	551.36 ± 6.64	<0.001*

UDVA= Uncorrected distance visual acuity; logMAR = logarithm of the minimum angle of resolution; Kmax = maximum keratometry; CCT=central corneal thickness;

*Paired Sample t-Test; ^#^ Wilcoxon Signed-Rank Test

**Fig. 2:**
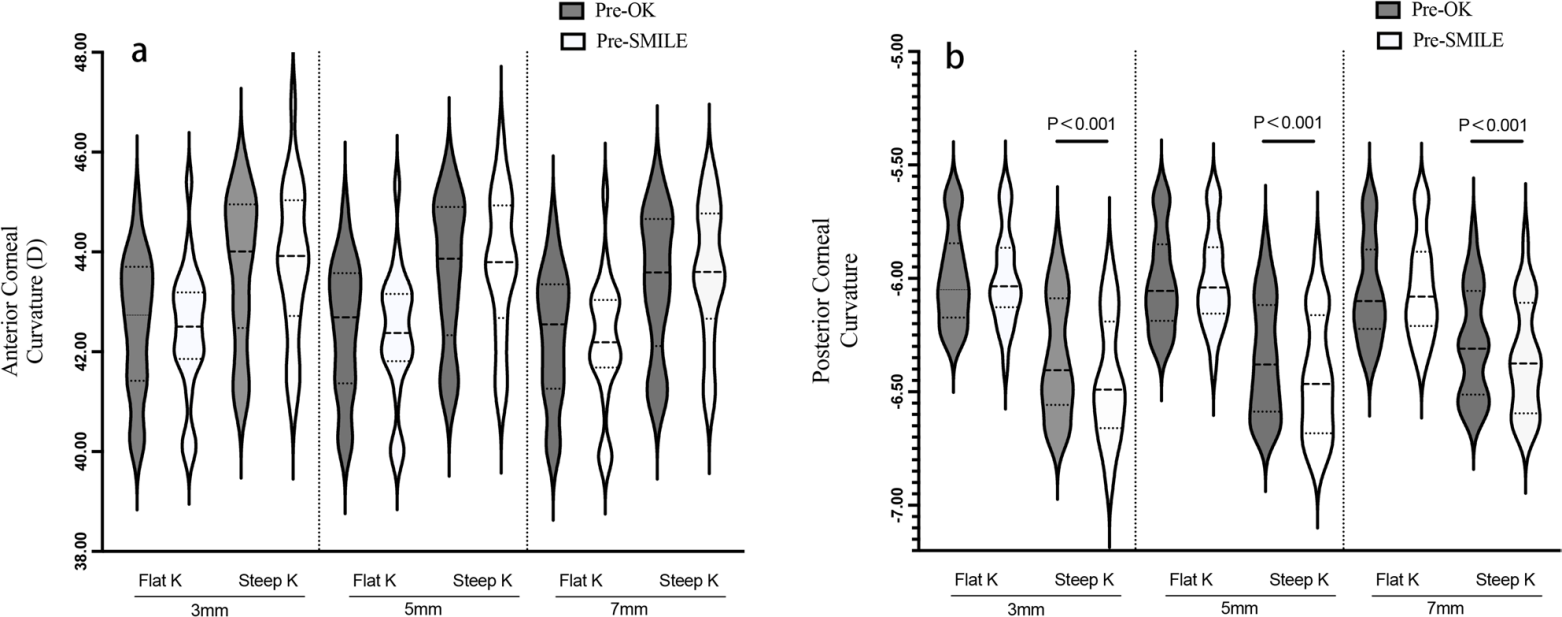
Statistical significance of the details on corneal parameters in the OK Group before OK treatment (Pre-OK) and before SMILE Surgery (Pre-SMILE). This violin plot illustrates the distribution of anterior (**a**) and posterior (**b**) corneal curvature measurements at 3 mm, 5 mm, and 7 mm before orthokeratology lens wear (Pre-OK) and before SMILE surgery (Pre-SMILE). The width of each violin reflects the relative frequency of curvature values. The upper and lower ends of each violin plot represent the maximum and minimum data points (excluding outliers). The upper and lower edges of the black lines indicate the third quartile (Q3) and first quartile (Q1), respectively, while the central line denotes the median curvature value. The posterior corneal curvature showed significant steepening, with steep K values decreasing by 0.09D, 0.08D, and 0.07D at the 3 mm, 5 mm, and 7 mm zones, respectively, from Pre-OK to Pre-SMILE, with all changes reaching statistical significance (Paired Sample t-Test, *P* < 0.001)

**Fig. 3:**
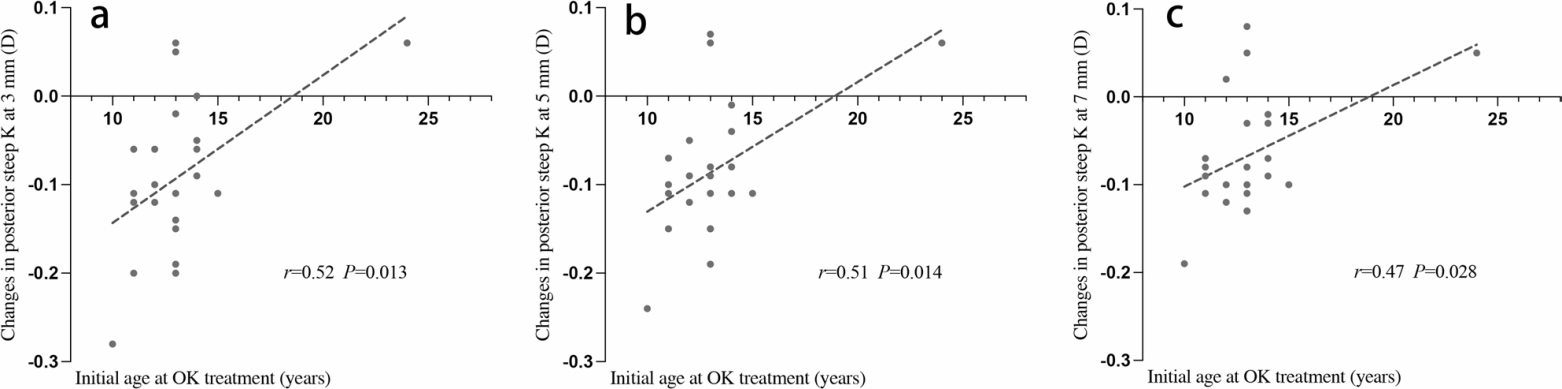
Correlation between initial age at OK treatment and changes in posterior steep K at the 3 mm (**a**), 5 mm (**b**), and 7 mm (**c**) zones following OK lens discontinuation. Pearson’s *r* and *P* values are provided for each zone. Dashed lines represent linear regression fits. All correlations were statistically significant (*P* < 0.050)

Anterior corneal curvature (flat and steep K values) showed no significant change after OK discontinuation (all *P* > 0.050). However, the posterior cornea became slightly steeper: Fig. 2 demonstrates that a statistically significant increase in posterior corneal keratometric power was observed in the 3 mm, 5 mm, and 7 mm zones after discontinuation of long-term orthokeratology lens wear, compared to pre-OK values (*P* < 0.001).There were significant positive correlations between the initial age at OK treatment and changes in posterior steep K values at the 3 mm (*r* = 0.52, *P* = 0.013), 5 mm (*r* = 0.51, *P* = 0.014), and 7 mm (*r* = 0.47, *P* = 0.028) zones after OK lens discontinuation (Fig. 3). Patients who started wearing OK lenses at a younger age showed a more significant decrease in posterior corneal K values, indicating a significant increase in posterior corneal keratometric power, with a more pronounced steepening of the posterior corneal surface. The duration of OK lens wear showed a negative correlation with changes in posterior steep K at the 3 mm (*r* = − 0.408, *P* = 0.060), 5 mm (*r* = − 0.423, *P* = 0.050), and 7 mm (*r* = − 0.418, *P* = 0.053) zones (all showing borderline significance). This suggests that longer OK lens wear may result in greater steepening of the posterior corneal surface after discontinuation, as indicated by a decrease in K values and an increase in keratometric power. However, the correlations between changes in posterior steep K values and the duration of lens discontinuation at the 3 mm, 5 mm, and 7 mm zones did not reach statistical significance.

### Post-SMILE visual and corneal outcomes

SMILE surgery improved uncorrected vision markedly in both groups. At 1 month postoperatively, mean UDVA was approximately − 0.09logMAR in the OK group and − 0.08logMAR in the control group, with no significant difference between groups (*P* = 0.870). Table 3 details the longitudinal outcomes. In the early postoperative period, Kmax was significantly higher in the OK group compared to the control group at the 1-month and 6-month follow-ups; however, this difference was no longer significant at the final follow-up. Other keratometric values (flat and steep K at various zones) did not significantly differ between groups at any postoperative time point (*P* > 0.050 for all comparisons). Post-SMILE corneal HOA increased in both groups but showed no significant differences between the OK and control eyes at 1 month, 6 months, or final follow-up (*P* > 0.050)​. At the final follow-up, no significant differences were observed between the OK and control groups in any visual acuity or corneal parameter (*P* > 0.050 for all comparisons)​.

**Table 3:** Clinical outcomes (Mean ± SD) before and after SMILE for the control and OK group

	*n*	OK group (Mean ± SD)	*n*	Control (Mean ± SD)	*P* Value (OK Group vs. Control Group)
UDVA (logMAR)					
Preoperative	22	1.02 ± 0.31	22	1.04 ± 0.04	0.747
1 month	22	-0.09 ± 0.04	22	-0.08 ± 0.04	0.872
6 month	14	-0.08 ± 0.05	21	-0.10 ± 0.04	0.796
Last available FU	10	-0.08 ± 0.06	15	-0.09 ± 0.05	0.930
CDVA (logMAR)					
Preoperative	22	-0.08 ± 0.04	22	-0.08 ± 0.04	1.000
1 month	22	-0.10 ± 0.01	22	-0.09 ± 0.01	0.530
6 month	14	-0.10 ± 0.01	21	-0.09 ± 0.01	0.565
Last available FU	10	-0.13 ± 0.02	15	-0.09 ± 0.01	0.063
Sphere (D)					
Preoperative	22	-4.43 ± 1.56	22	-4.23 ± 1.30	0.440
1 month	22	0.50 ± 0.19	22	0.32 ± 0.19	0.493
6 month	14	0.41 ± 0.23	21	0.23 ± 0.19	0.543
Last available FU	10	0.13 ± 0.28	15	0.33 ± 0.23	0.561
Cylinder (D)					
Preoperative	22	-0.82 ± 0.63	22	-0.63 ± 0.58	0.115
1 month	22	-0.40 ± 0.09	22	-0.19 ± 0.09	0.095
6 month	14	-0.38 ± 0.11	21	-0.26 ± 0.09	0.418
Last available FU	10	-0.43 ± 0.13	15	-0.35 ± 0.10	0.650
Flat K (D)					
Preoperative	22	42.35 ± 1.29	22	41.96 ± 0.93	0.220
1 month	22	38.07 ± 0.26	22	38.17 ± 0.23	0.792
6 month	14	38.25 ± 0.28	21	38.30 ± 0.23	0.894
Last available FU	10	38.04 ± 0.33	15	38.03 ± 0.27	0.981
Steep K(D)					
Preoperative	22	43.74 ± 1.43	22	43.16 ± 0.97	0.085
1 month	22	39.12 ± 0.28	22	39.00 ± 0.24	0.739
6 month	14	39.30 ± 0.29	21	39.05 ± 0.24	0.509
Last available FU	10	38.85 ± 0.35	15	38.76 ± 0.28	0.830
Corneal astigmatism (D)					
Preoperative	22	-1.38 ± 0.80	22	-1.19 ± 0.54	0.224
1 month	22	-1.03 ± 0.13	22	-0.83 ± 0.11	0.259
6 month	14	-0.94 ± 0.14	21	-0.76 ± 0.11	0.298
Last available FU	10	-0.81 ± 0.16	15	-0.73 ± 0.13	0.684
CCT (µm)					
Preoperative	22	551.36 ± 6.64	22	554.91 ± 6.64	0.706
1 month	22	447.63 ± 7.79	22	466.76 ± 6.80	0.066
6 month	14	466.71 ± 8.33	21	468.81 ± 6.80	0.846
Last available FU	10	464.40 ± 9.85	15	459.47 ± 8.04	0.699
Kmax (D)					
Preoperative	22	44.76 ± 1.78	22	44.17 ± 0.76	0.585
1 month	22	52.17 ± 0.89	22	49.00 ± 0.78	0.008
6 month	14	52.03 ± 0.95	21	49.09 ± 0.78	0.018
Last available FU	10	49.69 ± 1.13	15	49.45 ± 0.92	0.870
HOA (µm)					
Preoperative	22	0.44 ± 0.04	22	0.47 ± 0.04	0.616
1 month	22	0.74 ± 0.05	22	0.62 ± 0.04	0.061
6 month	14	0.65 ± 0.05	21	0.71 ± 0.04	0.399
Last available FU	10	0.76 ± 0.06	15	0.74 ± 0.05	0.732
Coma (µm)					
Preoperative	22	0.20 ± 0.04	22	0.26 ± 0.04	0.262
1 month	22	0.37 ± 0.05	22	0.38 ± 0.04	0.884
6 month	14	0.30 ± 0.05	21	0.41 ± 0.04	0.076
Last available FU	10	0.43 ± 0.06	15	0.44 ± 0.05	0.864
Trefoil (µm)					
Preoperative	22	0.17 ± 0.03	22	0.20 ± 0.03	0.551
1 month	22	0.23 ± 0.03	22	0.21 ± 0.03	0.666
6 month	14	0.20 ± 0.03	21	0.23 ± 0.03	0.592
Last available FU	10	0.32 ± 0.04	15	0.24 ± 0.03	0.122
Spherical aberration (µm)					
Preoperative	22	0.23 ± 0.08	22	0.22 ± 0.03	0.717
1 month	22	0.42 ± 0.03	22	0.32 ± 0.03	0.030
6 month	14	0.37 ± 0.04	21	0.36 ± 0.03	0.930
Last available FU	10	0.39 ± 0.04	15	0.40 ± 0.03	0.873
Horizontal Coma (µm)					
Preoperative	22	-0.07 ± 0.07	22	-0.12 ± 0.07	0.575
1 month	22	0.17 ± 0.08	22	0.07 ± 0.07	0.342
6 month	14	0.16 ± 0.08	21	0.07 ± 0.07	0.454
Last available FU	10	0.13 ± 0.10	15	0.17 ± 0.08	0.803
Vertical Coma (µm)					
Preoperative	22	0.08 ± 0.12	22	0.11 ± 0.03	0.467
1 month	22	0.19 ± 0.04	22	0.21 ± 0.03	0.675
6 month	14	0.13 ± 0.04	21	0.22 ± 0.03	0.073
Last available FU	10	0.26 ± 0.04	15	0.19 ± 0.04	0.221

UDVA= Uncorrected distance visual acuity; logMAR = logarithm of the minimum angle of resolution; CDVA=corrected distance visual acuity; Kmax = maximum keratometry; HOA= Higher-order aberration; CCT=central corneal thickness; D = diopter; FU = follow-up; SD = standard deviation;

Corneal wavefront aberrations were recorded over a 6-mm pupil diameter

General linear mixed models with Bonferroni correction

### Vector astigmatism analysis

Vector astigmatism analysis (Alpins method) was likewise comparable between the two groups, with no significant differences in target-induced, surgically induced, or remaining astigmatism vectors (all *P* > 0.050) (Table 4).

**Table 4:** Alpins astigmatism analysis using refraction at 1 month postoperatively

	*n*	OK group	Control	*P* Value (OK Group vs. Control Group)
TIA vector(D)	22	0.71 ± 0.56	0.55 ± 0.50	0.346^#^
SIA vector(D)	22	0.93 ± 0.61	0.67 ± 0.58	0.152*
Difference vector(D)	22	0.41 ± 0.27	0.33 ± 0.25	0.166^*#*^
Magnitude of error(D)	22	-0.22 ± 0.28	-0.12 ± 0.27	0.250^*#*^
Angle of error(°)	22	-4.74 ± 24.20	-11.82 ± 16.50	0.100^*#*^
Absolute angle of error(°)	22	13.39 ± 20.53	12.40 ± 16.05	1.000^*#*^
Correction index	22	1.24 ± 0.76	0.91 ± 0.75	0.165*

TIA=Target-induced astigmatism; SIA= Surgically induced astigmatism;

*Independent sample t-test; ^#^ Mann-Whitney U test

## Discussion

Our study provides new insights into corneal morphological changes after discontinuing long-term orthokeratology and addresses the implications for subsequent SMILE surgery. The findings have direct clinical relevance for managing patients who transition from OK lenses to refractive surgery.

Importance of corneal stabilization before surgery: Our study emphasizes that achieving stable corneal topography is critical prior to any refractive surgery in former OK wearers. In practice, this means enforcing a sufficient lens “washout” period and closely monitoring the cornea during that time. We selected a minimum 3-month discontinuation period, guided by the *China Refractive Surgery Expert Consensus* [26]. Shorter cessation periods (weeks to 1 month) have been shown to result in incomplete corneal and refractive regression [27, 28]. Numerous reports on long-term OK wear suggest that the cornea may take weeks or even months to fully recover. Barr et al. [29] found that after 6 to 9 months of OK wear, the refractive state did not fully recover even 72 h after discontinuing the lenses. Santodomingo-Rubido et al. [30] observed residual effects one week after two years of OK wear. A notable extreme case is from Kang and Swarbrick: after 13 years of OK use, they waited 408 days (over a year) to ensure complete corneal regularization before performing LASIK [31]​​. In fact, our results indicate that some subtle changes (such as slight steepening of the posterior corneal curvature) might continue beyond 3 months. When sufficient discontinuation and careful monitoring are provided, refractive surgery remains safe and feasible for former OK lens wearers. Kang et al. suggested that several factors may influence the required discontinuation time: Patients with higher myopic correction may need longer recovery periods; A longer total duration of OK lens wear may extend the time required for full corneal recovery; Older patients may experience slower regression of corneal parameters [31]​​. However, it should be noted that the studies by Queirós and Kang had small sample sizes, and their conclusions should be interpreted with caution [24, 31]. It is especially important to note that large-sample, long-term, high-quality longitudinal studies are still lacking. Therefore, current recommendations should be regarded as clinical references rather than absolute standards [32].

Consistent with previous studies, our findings confirm that overnight OK lens wear induces flattening of the central anterior corneal curvature—an effect that is largely reversible following lens discontinuation [18, 33]. However, the OK-induced increase in corneal astigmatism appeared to persist to some extent, even after at least a 3-month washout period [17]​. Interestingly, we also documented a small but statistically significant reduction in both corneal volume and CCT. While Zhang et al. [34] reported an increase in corneal volume during OK treatment—attributed to mild edema that resolved after lens removal—our long-term OK wearers exhibited a decrease in corneal volume post-discontinuation, which cannot be explained by residual edema alone. Given that our study demonstrated changes in CCT and corneal volume, future research should utilize epithelial thickness mapping via anterior segment optical coherence tomography (AS-OCT) to further elucidate the regional epithelial redistribution and biomechanical remodeling of the cornea associated with long-term OK lens wear. Indeed, while short-term OK use typically results in reversible changes such as mild epithelial thinning, stromal compression, and temporary edema [34, 35], extended wear is associated with more persistent alterations in corneal characteristics. Prior studies have shown that long-term OK users have reduced CCT compared to non-users [18–21, 33], and exhibit characteristic changes in epithelial thickness distribution—specifically, central epithelial thinning [33, 36] accompanied by relative mid-peripheral thickening [19, 33, 36, 37]. In our study, the average corneal thinning was relatively small (10 μm). Previous research has shown that the reduction in corneal thickness after OK lens wear in children is primarily attributable to epithelial thinning (approximately 80%) [38]. Therefore, this slight reduction in central corneal thickness can be considered clinically negligible in the context of refractive surgery, particularly regarding the eligibility for SMILE, as SMILE mainly concerns stromal tissue thickness. It is noteworthy that all eyes in our OK group maintained sufficient central corneal thickness to meet the safety thresholds for SMILE, and no intraoperative or postoperative complications were observed.

Posterior Corneal Curvature Changes: Our study is among the first to evaluate posterior corneal surface behavior following long-term OK wear. Interestingly, we observed a slight increase in posterior corneal steepness that persisted even after lens discontinuation. Specifically, the posterior corneal keratometric power showed a marginal steepening of less than 0.1 diopters in the OK group compared to the pre-OK baseline. Previous studies have reported mixed findings regarding posterior corneal changes induced by OK. Some short-term studies described transient posterior flattening during the initial week of lens wear, possibly reflecting acute biomechanical responses [39]. In contrast, medium [40] and long-term studies [41, 42] found no sustained changes in posterior curvature with continued OK use. For instance, Chen et al. [40] noted that posterior steepening observed after overnight wear resolved within hours of lens removal in adult subjects. Our findings differ in that a slight residual steepening persisted even after extended cessation of OK lenses. To further assess the clinical relevance of these findings, we examined correlations between posterior corneal curvature changes and variables such as baseline age, OK lens wear duration, and discontinuation period. We found that a younger age at the start of OK treatment (significantly) and longer lens wear (borderline) were associated with greater posterior corneal steepening. These results indicate that both younger initial age and longer lens wear may contribute to more pronounced posterior corneal keratometric power changes, which are not easily reversed after stopping lens use. We hypothesize a potential physiological mechanism: overnight OK wear may suppress the central corneal swelling that normally occurs during sleep, due to lens-induced pressure and tear film thinning beneath the lens. Meanwhile, the mid-peripheral cornea, not under direct compression, may still undergo slight nocturnal swelling. Over time, this differential edema pattern could lead to subtle redistribution of the posterior corneal contour, resulting in a slight backward displacement or steepening of the mid-peripheral posterior surface [40, 43]. Supporting this hypothesis, Alharbi et al. [43] demonstrated that overnight OK use significantly suppresses central stromal edema, potentially altering posterior corneal curvature. Importantly, the magnitude of posterior steepening observed in our cohort (< 0.1 D) was clinically negligible and well below the detection threshold of routine clinical examination. Moreover, it did not impact SMILE outcomes in any measurable way. Postoperatively, posterior curvature in eyes with prior OK wear was indistinguishable from that of control eyes. While statistically significant, this subtle change in posterior corneal shape does not appear to compromise refractive surgical safety or visual performance. However, the steepening of the posterior surface may still increase the risk of postoperative ectasia. Nonetheless, it represents a novel finding that contributes to our understanding of the biomechanical effects of long-term OK wear.

Multiple studies have reported an increase in higher-order aberrations (HOAs) [19, 44] during OK lens wear, including spherical aberrations (SAs) [44], coma, trefoil, and tetrafoil. Our study similarly found that long-term OK lens wear was associated with an increase in spherical aberration and a reduction in vertical coma. The increase in spherical aberration has been attributed to the cornea becoming more oblate after treatment [44]. Conversely, the reduction in vertical coma reflects a well-centered OK treatment zone, which reduces corneal asymmetry while increasing asphericity. Following SMILE, former OK eyes showed no significant difference in HOAs compared to control eyes. After complete corneal recovery, HOA levels become comparable between groups. Overall, former OK eyes achieve equally excellent visual quality after SMILE as eyes without prior OK wear.

Comparison of SMILE and LASIK in Patients with Prior OK lens: Queirós et al. [24] reported that LASIK performed after OK lens wear achieved outcomes comparable to those in control patients. Additionally, case reports—such as the 408-day washout case by Kang and Swarbrick [31]—support that LASIK outcomes are not compromised, provided that corneal stability is confirmed preoperatively. Our study builds on this knowledge by specifically evaluating SMILE outcomes in patients with a history of OK lens wear. Our results demonstrate that the outcomes of SMILE surgery were similarly unaffected, consistent with the principle that both SMILE and LASIK should be performed on stable corneas. Are there differences between LASIK and SMILE in terms of corneal response? The use of OK lenses induces changes in the intracellular fluid levels of corneal epithelial cells: a reduction of intracellular fluid in the central epithelium and an increase in the mid-peripheral epithelium, resulting in a characteristic pattern of central epithelial thinning and mid-peripheral epithelial thickening. This concept has been confirmed by histological studies from Choo et al. in cat models, which demonstrated that the thinning of the central corneal epithelium after OK lens wear is not due to migration or loss of epithelial cells but rather due to cellular compression causing intracellular fluid to shift toward the mid-periphery [45]. These changes may pose problems during LASIK surgery, as the epithelium-to-stroma ratio may vary across the 110-µm femtosecond laser flap. This can affect the flap’s integrity, increasing the risk of striae formation, and may also reduce the central residual corneal stroma thickness, leading to unpredictable ablation treatment and an increased risk of postoperative ectasia [32]. However, SMILE is a flapless procedure that only involves a smaller incision, and unlike LASIK, it does not carry flap-related complications. Still, the reduction in central residual corneal stroma thickness and the steepening of posterior corneal curvature could lead to unstable ablation and an increased risk of postoperative ectasia. In conclusion, based on our study and existing literature, both SMILE and LASIK demonstrate similar corneal and visual outcomes in previous OK lens wearers, as long as preoperative stability standards are met.

Despite the encouraging results, this study has several limitations. The retrospective design and relatively small sample size may restrict the generalizability of our findings. Additionally, we did not directly assess certain aspects of visual quality, such as contrast sensitivity or night vision symptoms, following SMILE surgery; instead, these parameters were inferred from higher-order aberrations (HOAs). Incorporating patient-reported outcomes in future research would provide a more comprehensive evaluation of visual quality. Another limitation is the lack of detailed analysis of corneal epithelial thickness and its regional variations. High-resolution imaging techniques, such as AS-OCT, could be employed in future studies to dynamically monitor structural changes in different corneal layers following OK lens wear and SMILE surgery. This would offer deeper insights into corneal remodeling mechanisms and their impact on postoperative refractive outcomes and visual quality. Furthermore, biomechanical assessments using devices such as the Corvis ST could provide valuable information on whether observed morphological changes are associated with biomechanical alterations. Prospective studies with larger cohorts are necessary to validate our findings and to assess the long-term stability of surgical outcomes in this patient population. In particular, investigating long-term outcomes (≥ 5 years post-SMILE) would be important to confirm whether patients with a history of OK lens wear experience the same stability and corneal health as typical SMILE patients. Given that these individuals undergo two corneal interventions in their lifetime (one temporary and one permanent), it remains to be seen whether any differences emerge over time in terms of refractive regression or ectasia rates. Our current findings suggest no increased risk, but longitudinal data are needed to substantiate this hypothesis.

This study demonstrates that discontinuation of long-term ok lens wear results in partial regression of associated corneal changes. While anterior corneal curvature and uncorrected visual acuity generally return to baseline, mild increases in astigmatism, subtle reductions in CCT and corneal volume, and steepening of the posterior corneal surface may persist. These residual changes may, in some cases, limit surgical eligibility and increase the risk of postoperative corneal ectasia. In our cohort, eyes with a history of OK lens wear achieved visual acuity, refractive outcomes, and corneal morphology comparable to those of control eyes without prior lens use. No additional intraoperative or postoperative complications were observed that could be attributed to previous OK wear. These findings support the notion that ok-induced corneal changes are largely reversible and that SMILE can be performed safely in appropriately selected and stabilized candidates. We emphasize the importance of confirming both corneal topographic and refractive stability through serial evaluations prior to surgical intervention. With careful patient selection and adequate preoperative timing, refractive surgery eligibility may be confidently extended to the growing population of individuals who previously underwent OK for myopia control. This sequential management strategy—orthokeratology in childhood followed by SMILE in adulthood for definitive refractive correction—offers a comprehensive, stage-based approach to myopia management that may contribute to improved long-term ocular health and patient quality of life.

## Data Availability

The data that support the findings of this study are available from the corresponding author upon reasonable request.

## Abbreviations

OK: Orthokeratology
SMILE: Small-Incision Lenticule Extraction
UDVA: Uncorrected Distance Visual Acuity
CDVA: Corrected Distance Visual Acuity
LogMAR: Logarithm of the Minimum Angle of Resolution
CCT: Central Corneal Thickness
Kmax: Maximum Keratometry
HOA(s): Higher-Order Aberration(s)
D: Diopter
SD: Standard Deviation
FU: Follow-up
TIA: Target-Induced Astigmatism
SIA: Surgically Induced Astigmatism

## References

[1] Wu PC, Huang HM, Yu HJ, Fang PC, Chen CT. Epidemiology of myopia. Asia Pac J Ophthalmol (Phila). 2016;5(6):386–93. 27898441 10.1097/APO.0000000000000236

[2] Sun MT, Tran M, Singh K, Chang R, Wang H, Sun Y. Glaucoma and myopia: diagnostic challenges. Biomolecules. 2023;13(3). 10.3390/biom13030562 PMC10046607 36979497

[3] Gabriel M, Großpötzl M, Wallisch F, Djavid D, Pregartner G, Haas A, et al. In-depth analysis of risk factors for pseudophakic retinal detachments and retinal breaks. Acta Ophthalmol. 2022;100(3):e694–700. 34258879 10.1111/aos.14974 PMC9290023

[4] Sun Y, Peng Z, Zhao B, Hong J, Ma N, Li Y, et al. Comparison of trial lens and computer-aided fitting in orthokeratology: a multi-center, randomized, examiner-masked, controlled study. Cont Lens Anterior Eye. 2024:102172. 10.1016/j.clae.2024.102172 38806329

[5] Sun J, Zhou J, Zhao P, Lian J, Zhu H, Zhou Y, et al. High prevalence of myopia and high myopia in 5060 Chinese university students in Shanghai. Invest Ophthalmol Vis Sci. 2012;53(12):7504–9. 23060137 10.1167/iovs.11-8343

[6] Lv L, Zhang Z. Pattern of myopia progression in Chinese medical students: a two-year follow-up study. Graefes Arch Clin Exp Ophthalmol. 2013;251(1):163–8. 22678717 10.1007/s00417-012-2074-9

[7] Logan NS, Bullimore MA. Optical interventions for myopia control. Eye (Lond). 2024;38(3):455–63. 37740053 10.1038/s41433-023-02723-5 PMC10858277

[8] Sankaridurg P, Berntsen DA, Bullimore MA, Cho P, Flitcroft I, Gawne TJ, et al. IMI 2023 digest. Invest Ophthalmol Vis Sci. 2023;64(6):7. 37126356 10.1167/iovs.64.6.7 PMC10155872

[9] Zhou J, Xue F, Zhou X, Naidu RK, Qian Y. Thickness profiles of the corneal epithelium along the steep and flat meridians of astigmatic Corneas after orthokeratology. BMC Ophthalmol. 2020;20(1):240. 32560640 10.1186/s12886-020-01477-y PMC7304131

[10] Zhao W, Wang J, Chen J, Xie H, Yang J, Liu K, et al. The rate of orthokeratology lens use and associated factors in 33,280 children and adolescents with myopia: a cross-sectional study from Shanghai. Eye (Lond). 2023;37(15):3263–70. 37046055 10.1038/s41433-023-02503-1 PMC10564736

[11] Shao W-Y, Jia H-Z, Cui B, Cao L-Q, Qin L-W, Zang G-M, et al. Analysis of corneal refractive surgery in soldiers from a specific region, China. 2020.

[12] Sharma VK, Sati A, Kumar S. Small incision lenticule extraction (SMILE) refractive surgery: our initial experience. Med J Armed Forces India. 2022;78(Suppl 1):S105–10. 36147395 10.1016/j.mjafi.2021.07.012 PMC9485860

[13] Lin MY, Tan HY, Chang CK. Myopic regression after FS-LASIK and SMILE. Cornea. 2024. 10.1097/ICO.0000000000003573 PMC11530297 38780430

[14] Ma L, Xu M, Wang J, Niu X. Analysis of the reasons for the discontinuation of orthokeratology lens use: A 4-Year retrospective study. Eye Contact Lens. 2022;48(8):335–9. 35877184 10.1097/ICL.0000000000000910 PMC9298146

[15] Nieto-Bona A, Gonzalez-Mesa A, Nieto-Bona MP, Villa-Collar C, Lorente-Velazquez A. Short-term effects of overnight orthokeratology on corneal cell morphology and corneal thickness. Cornea. 2011;30(6):646–54. 21282996 10.1097/ICO.0b013e31820009bc

[16] Chen X, Yang B, Wang X, Ma W, Liu L. The alterations in ocular biometric parameters following short-term discontinuation of long-term orthokeratology and prior to subsequent lens fitting: a preliminary study. Ann Med. 2023;55(2):2282745. 37988719 10.1080/07853890.2023.2282745 PMC10836244

[17] Chen Z, Zhou J, Xue F, Zhou X, Qu X. Increased corneal toricity after Long-Term orthokeratology lens wear. J Ophthalmol. 2018;2018:7106028. 30425855 10.1155/2018/7106028 PMC6218724

[18] Nieto-Bona A, Gonzalez-Mesa A, Nieto-Bona MP, Villa-Collar C, Lorente-Velazquez A. Long-term changes in corneal morphology induced by overnight orthokeratology. Curr Eye Res. 2011;36(10):895–904. 21950694 10.3109/02713683.2011.593723

[19] Reinstein DZ, Gobbe M, Archer TJ, Couch D, Bloom B. Epithelial, stromal, and corneal pachymetry changes during orthokeratology. Optom Vis Sci. 2009;86(8):E1006–14. 19584769 10.1097/OPX.0b013e3181b18219

[20] Huang Y, Li X, Ding C, Chen Y, Chen H, Bao J. Orthokeratology reshapes eyes to be less prolate and more symmetric. Cont Lens Anterior Eye. 2022;45(4):101532. 34736858 10.1016/j.clae.2021.101532

[21] Li F, Jiang ZX, Hao P, Li X. A Meta-analysis of central corneal thickness changes with overnight orthokeratology. Eye Contact Lens. 2016;42(2):141–6. 25828512 10.1097/ICL.0000000000000132

[22] Alharbi A, Swarbrick HA. The effects of overnight orthokeratology lens wear on corneal thickness. Invest Ophthalmol Vis Sci. 2003;44(6):2518–23. 12766051 10.1167/iovs.02-0680

[23] Nichols JJ, Marsich MM, Nguyen M, Barr JT, Bullimore MA. Overnight orthokeratology. Optom Vis Sci. 2000;77(5):252–9. 10831215 10.1097/00006324-200005000-00012

[24] Queiros A, Villa-Collar C, Amorim-de-Sousa A, Gargallo-Martinez B, Gutierrez-Ortega R, Gonzalez-Perez J, et al. Corneal morphology and visual outcomes in LASIK patients after orthokeratology: A pilot study. Cont Lens Anterior Eye. 2018;41(6):507–12. 30217386 10.1016/j.clae.2018.09.001

[25] van der Star L, Vasiliauskaite I, Oellerich S, Groeneveld-van Beek EA, Ghaly M, Laouani A, et al. Bowman layer onlay grafting as a minimally invasive treatment for the most challenging cases in keratoconus. Am J Ophthalmol. 2024;261:54–65. 37935272 10.1016/j.ajo.2023.10.004

[26] Group CLCRSPCTSEC, Association RGotOBotCEH. Chinese laser corneal refractive surgery preoperative corneal topography screening expert consensus (2024). Chin J Experimental Ophthalmol. 2024;42(12):1073–8.

[27] Soni PS, Nguyen TT, Bonanno JA. Overnight orthokeratology. Eye Contact Lens: Sci Clin Pract. 2004;30(4):254–62. 10.1097/01.icl.0000140637.58027.9b 15499266

[28] Hiraoka T, Okamoto C, Ishii Y, Okamoto F, Oshika T. Recovery of corneal irregular astigmatism, ocular higher-order aberrations, and contrast sensitivity after discontinuation of overnight orthokeratology. Br J Ophthalmol. 2009;93(2):203–8. 19019936 10.1136/bjo.2007.136655

[29] Barr JT, Rah MJ, Meyers W, Legerton J. Recovery of refractive error after corneal refractive therapy. Eye Contact Lens. 2004;30(4):247–51. discussion 63 – 4. 15499264 10.1097/01.icl.0000140234.85617.88

[30] Santodomingo-Rubido J, Villa-Collar C, Gilmartin B, Gutierrez-Ortega R. Short-term changes in ocular biometry and refraction after discontinuation of long-term orthokeratology. Eye Contact Lens. 2014;40(2):84–90. 24508773 10.1097/ICL.0000000000000014

[31] Kang P, Swarbrick H. Discontinuation of long term orthokeratology lens wear and subsequent refractive surgery outcome. Cont Lens Anterior Eye. 2017;40(6):436–9. 28712892 10.1016/j.clae.2017.07.001

[32] Wang VM, Moin KA, Hoopes PC, Moshirfar M. Corneal refractive surgery considerations in patients with history of orthokeratology. Eye Contact Lens. 2025;51(2):98–105. 39508787 10.1097/ICL.0000000000001138

[33] Haque S, Fonn D, Simpson T, Jones L. Corneal and epithelial thickness changes after 4 weeks of overnight corneal refractive therapy lens wear, measured with optical coherence tomography. Eye Contact Lens. 2004;30(4):189–93. discussion 205-6. 15499246 10.1097/01.icl.0000140223.60892.16

[34] Zhang YE, Ouzzani M, Wright C, Sorbara L. Changes in corneal thickness, corneal volume, and densitometry after long-term orthokeratology wear. Cont Lens Anterior Eye. 2023;46(1):101703. 35550858 10.1016/j.clae.2022.101703

[35] Lam AK, Wong YZ, Cheng SY. Corneal volume measures for monitoring contact lens induced corneal swelling: a pilot study. Clin Exp Optom. 2011;94(1):93–7. 20735785 10.1111/j.1444-0938.2010.00517.x

[36] Zhong X, Chen X, Xie RZ, Yang J, Li S, Yang X, et al. Differences between overnight and long-term wear of orthokeratology contact lenses in corneal contour, thickness, and cell density. Cornea. 2009;28(3):271–9. 19387227 10.1097/ICO.0b013e318186e620

[37] Lian Y, Shen M, Jiang J, Mao X, Lu P, Zhu D, et al. Vertical and horizontal thickness profiles of the corneal epithelium and bowman’s layer after orthokeratology. Invest Ophthalmol Vis Sci. 2013;54(1):691–6. 23221070 10.1167/iovs.12-10263

[38] Wan K, Yau HT, Cheung SW, Cho P. Corneal thickness changes in myopic children during and after short-term orthokeratology lens wear. Ophthalmic Physiol Opt. 2021;41(4):757–67. 33878198 10.1111/opo.12824

[39] Owens H, Garner LF, Craig JP, Gamble G. Posterior corneal changes with orthokeratology. Optom Vis Sci. 2004;81(6):421–6. 15201715 10.1097/01.opx.0000135097.99877.5d

[40] Chen D, Lam AK, Cho P. Posterior corneal curvature change and recovery after 6 months of overnight orthokeratology treatment. Ophthalmic Physiol Opt. 2010;30(3):274–80. 20444134 10.1111/j.1475-1313.2010.00710.x

[41] Tsukiyama J, Miyamoto Y, Higaki S, Fukuda M, Shimomura Y. Changes in the anterior and posterior radii of the corneal curvature and anterior chamber depth by orthokeratology. Eye Contact Lens. 2008;34(1):17–20. 18180677 10.1097/ICL.0b013e3180515299

[42] Stillitano IG, Chalita MR, Schor P, Maidana E, Lui MM, Lipener C, et al. Corneal changes and wavefront analysis after orthokeratology fitting test. Am J Ophthalmol. 2007;144(3):378–86. 17651677 10.1016/j.ajo.2007.05.030

[43] Alharbi A, La Hood D, Swarbrick HA. Overnight orthokeratology lens wear can inhibit the central stromal edema response. Invest Ophthalmol Vis Sci. 2005;46(7):2334–40. 15980219 10.1167/iovs.04-1162

[44] Lorente-Velazquez A, Madrid-Costa D, Nieto-Bona A, Gonzalez-Mesa A, Carballo J. Recovery evaluation of induced changes in higher order aberrations from the anterior surface of the cornea for different pupil sizes after cessation of corneal refractive therapy. Cornea. 2013;32(4):e16–20. 23132438 10.1097/ICO.0b013e318261eb66

[45] Choo JD, Caroline PJ, Harlin DD, Papas EB, Holden BA. Morphologic changes in Cat epithelium following continuous wear of orthokeratology lenses: a pilot study. Cont Lens Anterior Eye. 2008;31(1):29–37. 17913568 10.1016/j.clae.2007.07.002

